# Superhydrophobicity Effects on Spheroid Formation, Structure, and Viability on Co-Culture Conditions

**DOI:** 10.3390/ph18070953

**Published:** 2025-06-24

**Authors:** María del Carmen Morán, Francesca Cirisano, Michele Ferrari

**Affiliations:** 1 Departament de Bioquímica i Fisiologia, Secció de Fisiologia, Facultat de Farmàcia i Ciències de l’Alimentació, Universitat de Barcelona, Avda. Joan XXIII, 27-31, 08028 Barcelona, Spain; 2 Institut de Nanociència i Nanotecnologia—IN^2^UB, Universitat de Barcelona, Avda. Diagonal, 645, 08028 Barcelona, Spain; 3 CNR-ICMATE—Istituto di Chimica della Materia Condensata e di Tecnologie per l’Energia, Via De Marini, 6, 16149 Genova, Italy; francesca.cirisano@cnr.it

**Keywords:** 3D profilometry, co-culture, spheroids, superhydrophobicity, surfaces, viability

## Abstract

**Background/Objectives:** Three-dimensional (3D) cell culture models more accurately simulate the in vivo tissue environments as compared to conventional two-dimensional (2D) monolayer cultures. Among these, spheroid cultures are particularly valuable for pharmaceutical research, as they allow for the study of tumor growth, drug responses, and cell–cell interactions in a physiologically relevant manner. Superhydrophobic surfaces (SHSs) have shown a promise in enhancing spheroid formation by reducing cell–substrate adhesion and promoting cell–cell aggregation. This study aims to evaluate the effectiveness of two different SHS coatings (SHS1: fluorinated; SHS2: silicone-based) in generating co-culture spheroids composed of non-tumoral fibroblasts (3T3) and tumoral epidermoid carcinoma cells (A431), thereby mimicking aspects of the tumor microenvironment. **Methods:** Co-cultures of 3T3 and A431 cells were seeded at varying ratios onto SHS1 and SHS2 substrates to assess their ability to support 3D spheroid formation. Spheroids were characterized by measurements of circularity and size distribution, viability through live/dead staining, and surface topography using 3D profilometry. **Results:** Spheroid formation was significantly influenced by both the surface properties and the fibroblast-to-carcinoma cell ratio. The fluorinated SHS1 surface facilitated superior cell viability and promoted the formation of well-rounded, uniform spheroids. In contrast, the silicone-based SHS2 surface resulted in less defined spheroidal structures and lower overall viability. Profilometry confirmed more consistent and compact 3D architectures on SHS1. **Conclusions:** This study demonstrates that SHS1, a fluorinated superhydrophobic coating, is more effective than SHS2 in supporting the formation of viable and structurally coherent 3D co-culture spheroids. These findings underscore the potential of SHS1 as a low-cost, tunable platform for developing in vitro cancer models and advancing the study of tumor–stroma interactions.

## 1. Introduction

Three-dimensional (3D) cell culture models have emerged as powerful tools to better mimic the in vivo microenvironment of tissues and tumors. Among these, spheroid cultures provide a physiologically relevant system for studying cell–cell interactions, drug responses, and tumor progression. Unlike traditional two-dimensional (2D) monolayer cultures, spheroids allow for the formation of complex cellular architectures, gradients of nutrients and oxygen, and more realistic cell signaling pathways [1,2,3,4,5,6]. Spheroids are increasingly used in preclinical oncology studies [7,8,9] due to their uniformity and accuracy in drug test findings.

Different techniques aim to produce spheroids, and in particular, microfluidic technology is developed to accurately place tumor spheroids in a hydrogel while mechanically probing the growing spheroids and the surrounding matrix. The growth and remodeling of tumors depend on the material characteristics of cancer cells and their matrix. Localized tumor growth occurs in soft hydrogels, along with changes in the Young’s modulus of the matrix. Tumor growth biomechanics involve cancer cells pushing against the surrounding matrix, creating mechanical resistance [10,11]. Microfluidic-based biochips offer more control over spheroid size, but decreasing cell adherence to the surface is a significant issue. Researchers applied antifouling coating materials, BSA and Pluronic F-68, to PMDS microfluidic biochip surfaces, resulting in on-chip homogeneous and uniform spheroids. This approach also prevented cell attachment and encouraged self-aggregation.

Superhydrophobic (SH) surfaces have emerged as a promising tool in biomedical research of the formation of three-dimensional (3D) spheroids. These surfaces, characterized by their extreme water repellency, minimize cell–surface interactions, thereby promoting cell–cell adhesion essential for spheroid formation. Moreover, such surfaces can be produced by easy and low-cost methods independently of the substrate. Compared to conventional techniques like hanging drop, liquid overlay, and microfluidic, SH surfaces offer a simpler and more reproducible approach to spheroid formation. They facilitate the creation of spheroids without the need for specialized equipment or complex procedures, making them accessible for various laboratory settings [12,13].

In accordance with the above [14,15], the substrate and surface features have been studied to investigate their influence on cells sticking to surfaces. This affects the cell processes like spreading, migration, and differentiation in biological uses. Cell cultures allow for research on cell biology, tissue structure, disease, medication effects, and tissue engineering. New methods for growing 3D cell groups on low-wettability surfaces improve in vitro studies.

Common cell therapies aim to regenerate tissue by increasing cell numbers or addressing functional gaps. Monodispersed cells are often injected for incorporation, but this can lead to issues like cell death and damage and, moreover, not efficient recovery and economic, lab-friendly production still limit the widespread use of spheroids. To overcome this bias, a superhydrophobic coating was applied to glass surfaces for the 3D spheroid production of fibroblasts and keratinocytes. The spheroid production and retrieval in 2D culture were assessed. 3D profilometry evaluated the morphology of migrating cells, showing enhanced adhesion and proliferation [16,17].

A tunable superhydrophobic array device (SHArD) was designed in [18] to cultivate a therapeutically relevant CTC cluster model. The device was used to cultivate immortalized cancer cell lines in various sizes. The clusters showed higher expressions of E-cadherin and were more resistant to high fluid shear stress, suggesting that clustering may offer a metastatic advantage. The biochemistry of CTCs is still unclear. In this case [19] the use of 3D cell culture in cancer research can improve understanding and facilitate drug development. Traditional techniques have limitations due to smaller drop volumes. A new method involves creating superhydrophobic paper cones that hold large amounts of culture media, are resistant to bacteria, sterile, and autoclavable. This allows for the production of sizable ovarian cancer cell spheroids and drug screening for carboplatin effects.

A critical factor in the success of spheroid models is cell viability, which is influenced by cell composition, size, and microenvironmental conditions. As spheroids grow, oxygen, nutrients, and waste diffusion create gradients, leading to necrotic cores, especially in larger spheroids. The inclusion of non-tumoral cells (e.g., fibroblasts, immune cells) affects these viability patterns by modulating extracellular matrix production, cytokine signaling, and metabolic support [20].

The authors have previously investigated [15] the influence of substrate and surface properties of a material on cell adhesion, impacting processes like spreading, migration, proliferation, and differentiation. Cell cultures are used for studying cell biology, tissue architecture, disease processes, medication action, and tissue engineering. Recent methods for growing 3D cell aggregates with low-wettability surfaces provide consistent conditions for studying tumor and non-tumor cell lines, aligning cell biology with novel materials. In another study [16] the present authors explore the use of 3D spheroids for cell production, addressing drawbacks such as cell death, inadequate retention, and injury. A superhydrophobic coating was applied to glass surfaces, and the spheroids were produced and retrieved in 2D culture. 3D profilometry was used to evaluate the morphology of migrating cells, revealing enhanced adhesion and proliferation of the cells.

In recent years, co-culture spheroid models incorporating both tumoral and non-tumoral cell lines have gained significant attention due to their ability to simulate the heterotypic interactions present in the tumor microenvironment. These models can include various stromal, immune, or endothelial cells alongside cancer cells, reflecting the complexity of real tumors. The cellular ratio within these co-culture systems plays a crucial role in determining the structural and functional characteristics of the spheroids, influencing proliferation, invasion, and response to therapeutics [21,22].

The present study investigates the preparation of spheroid-based co-culture systems involving the squamous cell carcinoma (A431) cell line and the Murine Swiss albino fibroblasts (3T3 cell line) at different ratios on two different superhydrophobic surfaces. By varying the composition of these spheroids, we aim to assess how the cell ratio affects spheroid formation, physicochemical properties such as size and circularity and final viability. Understanding these interactions is essential for improving preclinical tumor models and developing more effective therapeutic strategies.

## 2. Results

### 2.1. Surface Characterization of Superhydophobic Coatings

**Figure 1:**
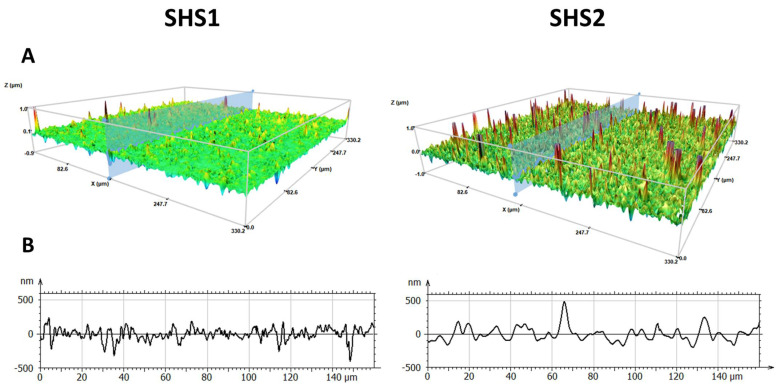
False color 3D views of SH surfaces (**A**) and the roughness profile (**B**) taken from each surface by interferometric and confocal profilometer at 20×. Fluoropolymer/silica (SHS1): average roughness, Sa = 65 nm; silicone/silica (SHS2): average roughness, Sa = 145 nm.

The superhydrophobic (SH) coatings have been deposited by spray technique using two different dispersions (see materials and methods) and studied focusing on their wettability, surface geometry and roughness (Sa). The two coated surfaces showed high contact angles (CA), higher than 160° with low hysteresis (<5°), resulting in drops rolling off the surface due to the low surface energy of the components: 11 mN/m for the fluorinated sample (SHS1) [23] and 24 mN/m for the silicone-based (SHS2) [24]. Both surfaces exhibit low wettability across the entire surface suggesting that superhydrophobicity was homogeneously distributed in the samples. This observation was confirmed by topography studies carried out by 3D confocal and interferometric profilometry (Figure 1). It was possible to observe that both SH coatings appeared to have a double scale nanometric roughness, but with some difference. Whereas in the fluoropolymer/silica coating (SHS1), the presence of peaks is low, with an average roughness of 65 nm, and the silicone/silica coating (SHS2) showed an average roughness of 145 nm, with the presence of numerous micrometric peaks.

### 2.2. Cell Behavior in SHS Substrates

Superhydrophobic surfaces have emerged as a promising tool in the formation of 3D cell spheroids. Previous works in our lab have demonstrated that these highly water-repellent surfaces can effectively promote the aggregation of various mammalian cell lines, including fibroblasts (3T3 cell line), keratinocytes (HaCaT cell line), tumoral cells (MCF-7 cell line), and macrophages (RAW 264.7 cell line) [16,25,26]. The superhydrophobic coatings, characterized by their extreme water repellency, minimize cell adhesion to the substrate, thereby encouraging cells to adhere to each other and form spheroids. This method not only facilitates spheroid formation but also allows for the easy recovery and subsequent 2D culture of the cells, maintaining their viability and functionality. Moreover, the use of SH surfaces has been linked to beneficial cellular responses. Thus, in the case of spheroids from representative skin cell lines such as 3T3 fibroblasts and HaCaT keratinocytes, the results demonstrated that SHS-induced spheroids promote enhanced cell–cell interactions, particularly at higher initial cell densities. The migrated cells from these spheroids exhibited morphological changes consistent with improved adhesion and proliferative capacity, highlighting the potential of these SHS-derived 3D cultures for skin tissue engineering and regenerative applications [16]. Furthermore, RAW264.7 macrophages cultured on these surfaces exhibited a polarization towards the M2 phenotype, which is associated with anti-inflammatory and tissue repair functions. Such findings underscore the potential of superhydrophobic surfaces in regenerative medicine and tissue engineering applications [26].

**Figure 2:**
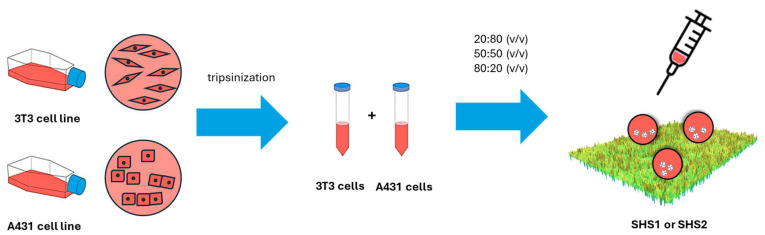
Schematic representation of 3T3 and A431 co-culture spheroid formation on superhydrophobic surfaces (either SHS1 or SHS2).

Considering these findings, the potential of the two proposed superhydrophobic surfaces (SHSs) for generating 3D spheroid cultures under co-culture conditions of tumoral and non-tumoral cells was explored for the first time in our lab. Co-culturing these two cell types within spheroids is essential for replicating the complex cellular interactions characteristic of the tumor microenvironment, which is composed not only of cancer cells but also of various stromal cells [27]. Testing the efficiency of the SHSs to create 3D spheroids under co-culture conditions was assayed using representative skin cell lines with tumoral and non-tumoral characteristics (Figure 2). 3T3 cells, derived from mouse fibroblasts, provide a supportive dermal matrix that mimics the skin’s connective tissue environment. A431 cells, derived from human epidermoid carcinoma, are often used to investigate cancer biology and the effects of tumor–stromal interactions. In this work, the non-tumoral 3T3 cell line and the tumoral A431cell line were mixed at three different cell ratios (20:80; 50:50, and 80:20) at an initial cell density of 2 × 10^6^ cell/mL over 24 h and 48 h incubation times.

**Figure 3:**
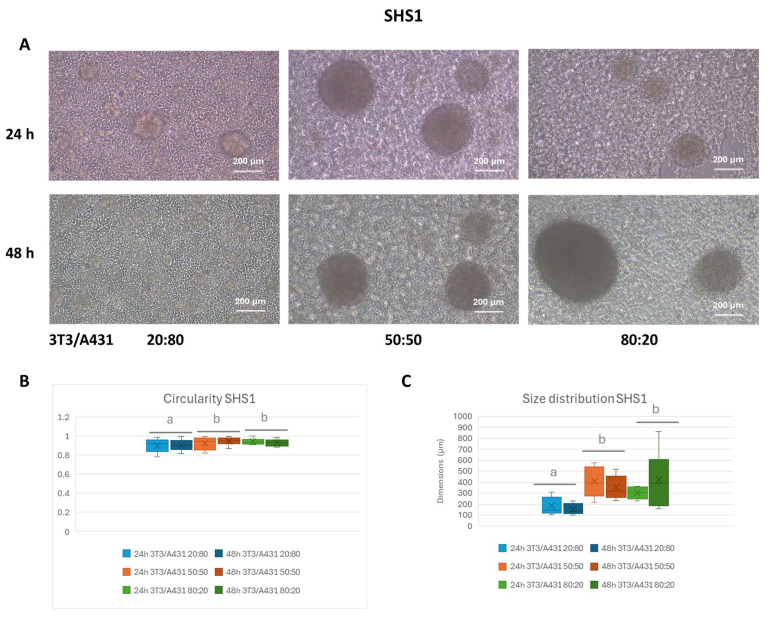
Phase contrast images (**A**) and box plots of circularity (**B**) and size distribution (**C**) under co-culture conditions in SHS1 after 24 and 48 h of incubation at three different cell ratios. The box includes the mean, median, and quartile calculation (exclusive median) of at least 10 individual spheroids for each condition. Results expressed as mean ± SEM were analyzed by two-way ANOVA using the factors time incubation (24 h, 48 h) and 3T3/A431 ratio composition (20:80, 50:50, and 80:20). Mean values without a common letter differ; *p* < 0.05.

**Figure 4:**
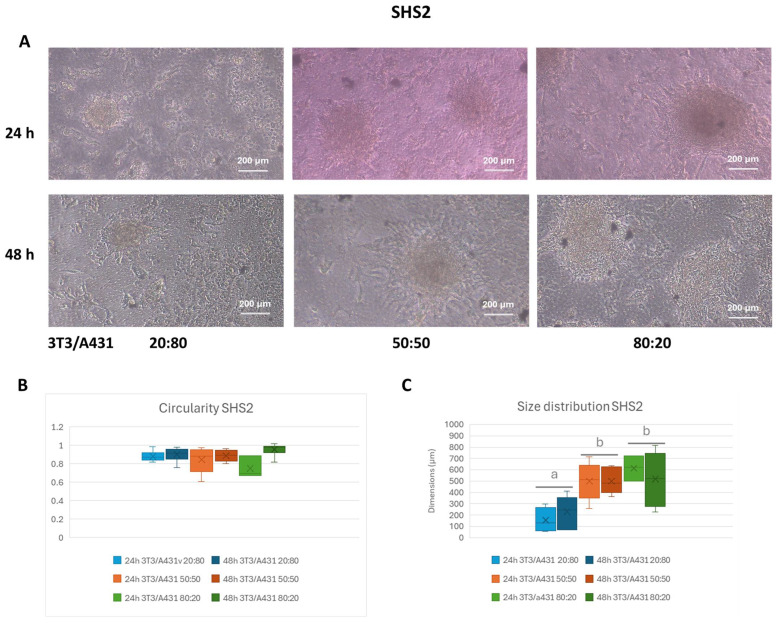
Phase contrast images (**A**) and box plots of circularity (**B**) and size distribution (**C**) on co-culture conditions in SHS2 after 24 and 48 h of incubation at three different cell ratios. The box includes the mean, median, and quartile calculation (exclusive median) of at least 10 individual spheroids for each condition. Results expressed as mean ± SEM were analyzed by two-way ANOVA using the factors time incubation (24 h, 48 h) and 3T3/A431ratio composition (20:80, 50:50, and 80:20). Mean values without a common letter differ; *p* < 0.05.

Both surfaces are very transparent, enabling the direct observation of cell aggregates with optical microscopy. The formation and morphology evolution of 3T3/A431 co-culture spheroids were assessed using brightfield microscopy. From the obtained images, further analyses of circularity and size distribution were performed. The results demonstrated that the characteristics of the formed spheroids were highly dependent on the cell ratios and the SH coatings (Figure 3 and Figure 4). When considering SHS1, the images illustrate the spheroid formation across all ratios, with marked differences in compactness and size (Figure 3A). Although the incubation time influences the final characteristics of the formed spheroids, the presence of a higher proportion of fibroblast cells suggested enhanced aggregation and stabilization. Thus, by 48 h, especially at the 80:20 ratio, spheroids appear significantly larger and more compact.

The circularity of cell aggregates, as a measure of the effectiveness of 3D spheroid formation, remained consistently high (>0.9) across all ratio and time points, indicating well-rounded spheroid morphology with minimal irregularities (Figure 3B). A slight increase in circularity from 24 to 48 h suggests improved structural organization over time, with significant differences (*p* < 0.05), for all the assayed cell ratios. When considering the effect of cell ratio, the highest circularity corresponds to the system at the 80:20 ratio. Significant differences (*p* < 0.05) were found between the 20:80 system and the other cell ratios.

Moreover, the size distribution data offer additional information into spheroid homogeneity (Figure 3C). Most spheroids in the 20:80 condition remained below 200 µm, either after 24 h or 48 h of incubation, with significant differences (*p* < 0.05) in comparison with other cell ratios, indicating delayed or inefficient aggregation. For the other cell ratio compositions (50:50 and 80:20 ratios), the generated spheroids remained in the 300–400 µm range, which differs significantly as a function of the incubation time (*p* < 0.05). Interestingly, the 50:50 ratio produced a more balanced size distribution, suggesting a transitional behavior between the low and high fibroblast ratio.

Using the SHS2 coating, spheroid formation was generally less robust and more heterogeneous compared to SHS1 (Figure 4A), with aggregates exhibiting poorly defined boundaries. Between the 24 h and 48 h time points, an increase in aggregation and compactness was observed, particularly in the 80:20 ratio, where spheroids appeared larger and more defined. Nonetheless, many structures retained an irregular morphology, indicating incomplete or unstable compaction.

The quantitative analysis reflects this morphological irregularity, with circularity values ranging between 0.75 and 0.95, as a function of cell ratio and incubation time (Figure 4B). At 24 h, circularity values were relatively high (~0.85–0.9) in the 20:80 and 50:50 ratio but notably lower (0.75) in the 80:20 condition, suggesting increased variability in spheroid shape at this ratio. At 48 h, the 80:20 ratio slightly improved their circularity (0.95), indicating some compaction over time. Statistical analyses demonstrated no significant differences as a function of either cell ratio composition or incubation time.

Size distribution analysis demonstrated that the spheroid development in SHS2 is also highly dependent on the cell ratio and time (Figure 4C). For the 20:80 composition, most spheroids remained under 200 µm, moving to the highest proportion of larger spheroids (>500 µm) for the 50:50 and 80:20 ratios, with significant differences (*p* < 0.05) in comparison with the lowest fibroblast content ratio. In all cases, significant differences (*p* < 0.05) were found when the different incubation times were compared.

### 2.3. Cell Viability on SHS Spheroids

A critical factor in the success of spheroid models is cell viability, which is influenced by cell composition, size, and microenvironmental conditions. Studies have shown that small spheroids (<200 µm) tend to maintain better viability, making them suitable for high-throughput drug testing. However, as spheroids grow, oxygen, nutrients, and waste diffusion create gradients, leading to necrotic cores, especially in larger spheroids. In addition, results have demonstrated that the inclusion of non-tumoral cells, such as fibroblasts or immune cells, affects these viability patterns by modulating extracellular matrix production, cytokine signaling, and metabolic support [28].

**Figure 5:**
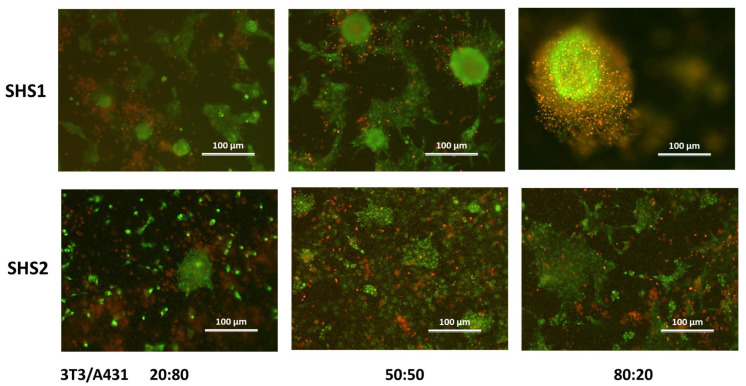
Representative fluorescence microscopy images of 3D spheroids formed on SHS1 and SHS2 with co-cultures of non-tumoral 3T3 and tumoral A431 cells at varying ratios (20:80, 50:50, and 80:20) after 48 h. Green fluorescence indicates AO stain in the live cells and red fluorescence indicates the EtBr stain in the dead cells. Scale bars represent 100 μm.

In this study, acridine orange/ethidium bromide (AO/EB) double staining was used to confirm the uniform distribution of viable cells within the spheroids formed by SH coatings. Figure 5 shows representative AO/EB fluorescence microscopy images of spheroids formed at 48 h of incubation. The observed staining patterns likely correspond to live (green) and dead (red) cells, providing insight into spheroid viability, morphology, and spatial cell distribution through the assayed conditions.

The SHS1 surface provided an excellent environment for spheroid formation, depending on the co-culture ratios. At both 20:80 and 50:50, distinct, round, compact spheroids are visible with high green fluorescence, indicating excellent viability and active cell–cell communication. The 80:20 condition showed larger, structured spheroids with minimal red signal, suggesting that even a lower proportion of A431 can integrate well within a fibroblast-rich environment on SHS1.

When considering the SHS2 surface, a more limited spheroid integrity can be observed. Spheroids appeared fragmented and loosely compact, with a prominent red signal across all ratios. For the 20:80 and 50:50 ratios show scattered cells and small clusters, indicating poor cell–cell cohesion and possible apoptotic stress. The 80:20 ratio displays slightly more organized green structures but still lacks the tight morphology characteristic of healthy spheroids. These observations suggest that 3T3 fibroblasts may not form strong adhesive or interactive networks with A431 cells on SHS2.

Table 1 summarizes the main characteristics of the spheroids generated in SHS1 and SHS2 assessed by both brightfield and fluorescence microscopies.

### 2.4. Morphological Parameters Determined by 3D Profilometry Analysis

**Figure 6:**
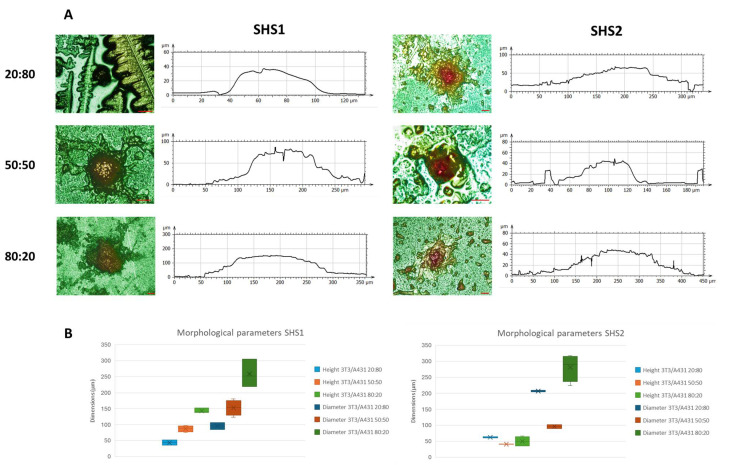
Representative 3D profilometry images in confocal mode (magnification 20×) of spheroids on co-culture conditions in SHS1 and SHS2 after 48 h of incubation at three different cell ratios, deduced by the corresponding profile sections. Scale bars represent 50 μm (**A**). Box plots of the deduced height and diameter dimensions from 3D profilometry profiles (**B**). The box includes the mean, median, and quartile calculation (exclusive median) of at least 10 individual spheroids for each condition Results expressed as mean ± SEM were analyzed by two-way ANOVA using the factors parameter (height, diameter) and 3T3/A431ratio composition (20:80, 50:50, and 80:20). Mean values without a common letter differ; *p* < 0.05.

Further insights into the surface analyses on the 3D spheroids generated in SHS1 and SHS2 were assessed using 3D Optical profilometry. Figure 6A shows representative 3D profilometry images and their corresponding surface height profile, enabling assessment of surface roughness, feature geometry, and topographical irregularities of the samples. Three-dimensional imaging revealed the detailed morphology of the 3D spheroids formed on SHSs. Thus, in the case of SHS1, they appeared highly symmetrical and compact, with well-defined central features and smooth surrounding areas. Moreover, the corresponding profile analysis for SHS1 samples exhibited more rounded peaks and gradual slopes, suggesting uniform growth. By comparison, SHS2 promoted the generation a more irregular and complex 3D morphologies, with heterogeneous textures surrounding the central elevated regions with steeper slopes.

The dimensional characteristics of 3T3/A431 co-culture spheroids formed at various cell rations on SHS1 and SHS2 were characterized based on the deduced height and diameter dimensions (Figure 6B). Both SHS1 and SHS2 spheroids exhibited significant differences in morphology depending on the co-culture ratio, with noticeable trends in spheroid size. For SHS1, the increase in 3T3 cell proportion correlates with a progressive increase in both height and diameter, with significant differences (*p* < 0.0001) between both parameters for the same composition cell ratio, and between cell ratio values. At the 80:20 ratio, the spheroids reached their maximum dimensions confirming that a higher fibroblast content promotes more robust and expansive spheroid growth.

In the case of SHS2, the influence of cell ratio compositions on dimensions is not so clear. The spheroid diameter seems to increase in the presence of more 3T3 cells, peaking at the 80:20 ratio. Significant differences (*p* < 0.05) were found for all the cell ratio compositions. However, the height does not show a corresponding rise. In fact, the highest spheroid height occurs at the 20:80 ratio and then decreases slightly at higher 3T3 proportions. Significant differences (*p* < 0.05) between height values at the different cell ratio compositions were found. This discrepancy suggests that SHS2 spheroid growth may be more planar or spread out in structure, as can also be deduced from the brightfield (Figure 4) and fluorescence (Figure 5) images, potentially due to differences in cell–cell interaction dynamics or changes in cell adhesion and compaction behavior.

The stark increase in diameter at the 80:20 ratio for SHS types suggests that 3T3 fibroblasts play a crucial role in determining lateral growth. However, the different height responses suggest that SHS1 and SHS2 might induce different spatial organization mechanisms or different cellular signaling profiles within the 3D culture environment. For instance, in SHS2 samples, the height remains relatively constant across all ratios, whereas in SHS1 samples, a higher percentage of fibroblasts cells appears to enhance substrate adhesion, influencing overall morphology.

## 3. Discussion

Cancer is a leading cause of death worldwide, with around 90% of fatalities resulting from metastasis. Advanced in vitro models like 3D tumor spheroids better mimic the tumor microenvironment than 2D cultures, enabling more accurate study of cancer progression and therapy response [29]. Superhydrophobic surfaces (SHSs) have emerged as a promising tool in biomedical research for facilitating the formation of 3D spheroids. These surfaces, characterized by their extreme water repellence, minimize cell–surface interactions, thereby promoting cell–cell adhesion essential for spheroid formation [15]. The high water repellence of SH surfaces causes culture media to form rounded droplets, compelling suspended cells within these droplets to aggregate due to limited attachment points. This environment promotes the formation of uniform and viable spheroids, as cells preferentially adhere to each other rather than to the substrate [30]

While SHSs present numerous benefits, certain challenges remain. Ensuring consistent coating homogeneity and durability of SH surfaces is crucial for reproducible results. Moreover, the homogeneity of surface coatings plays a critical role in defining their functional performance, particularly in applications requiring uniformity in thickness, adhesion, and surface properties. In this work, the 3D surface topography and cross-sectional profiles of SHS1 and SHS2 provide valuable information into the distribution characteristics of these coatings at the nanoscale (Figure 1). The cross-sectional profiles of SHS1 revealed a narrow amplitude range, maintaining fluctuations mostly within ±250 nm. This supports the observation of uniformity, reflecting a smooth, homogeneous film. In the case of SHS2, a few sharp spikes with amplitude variations reaching nearly ±500 nm could be observed, inducing certain heterogeneity in comparison with SHS1.

Although most studies in the literature have used spheroids composed of a single tumor cell type, monoculture spheroids are unable to replicate the complexity of tumors. Consequently, 3D tumor spheroid models with different levels of complexity of heterotypic (co-culture) spheroids have been developed [31]. Among the different cells, fibroblasts are crucial components of the tumor stroma due to their capability to modulate tumor progression and the sensitivity of cancer cells to treatment. Accordingly, the presence of stroma components should be considered in the modeling of tumor research. Based on the reported data, in this work the generation of spheroids was projected using co-culture ratios between the Murine Swiss albino fibroblasts (3T3 cell line), and the squamous cell carcinoma (A431) cell line.

Additionally, optimizing cell ratios is crucial for achieving uniform spheroid sizes. The effect of both incubation time and cell ratio compositions on the morphology, circularity, and size distribution of the spheroids generated in SHS1 and SHS2 was evaluated (Figure 3 and Figure 4 and Table 1). As a general trend, the circularity of cell aggregates, as a measure of the effectiveness of 3D spheroid formation, remained consistently high across all ratios, indicating well-rounded spheroid morphology with minimal irregularities in the case of spheroids prepared in SHS1 (Figure 3). However, SHS2 promoted the formation of aggregates with poorly defined boundaries (Figure 4). In both SHSs, a slight increase in circularity from 24 to 48 h suggested improved structural organization over time. When considering the effect of cell ratio, the shift toward larger spheroids at higher fibroblast content (80:20 ratio) underscores the role of fibroblasts in promoting spheroid growth and mass accumulation. These results agree with previous studies about the impact of fibroblasts presence on co-culturing tumor cells and fibroblasts, observing that co-cultured cells exhibited increased viability over time, suggesting that fibroblasts contribute to enhanced tumor cell survival [32]. When researchers developed stroma-rich multicellular tumor spheroids, the presence of stromal cells, such as fibroblasts, contributes to maintaining tumor cell viability within the spheroids [8].

The viability of spheroids is also strongly influenced by the ratio of tumoral and non-tumoral cells. As a general trend, for a higher tumoral cell ratio, lower overall viability can be expected either by the rapid proliferation of cancer cells or by the lack of a supportive extracellular matrix (ECM) leading to weaker structural integrity. For a balanced tumor–stromal ratio, a higher viability due to paracrine signaling and ECM production from stromal cells, which provide mechanical and biochemical supports. A higher stromal cell ratio may lead to reduced tumor cell proliferation and increased differentiation, but overall better spheroid stability and longer-term viability [28]. In this work, live/dead staining (Figure 5) has demonstrated that 3T3 fibroblasts seem to provide mechanical support and ECM components facilitating spheroid cohesion, which is better expressed on the SHS1 surface due to its non-adhesive, low-wettability nature that encourages self-aggregation. Moreover, in both SHSs, the 50:50 ratio supported the best balance between viability and structure, possibly due to optimal heterotypic cell–cell interactions. Interestingly, the cell growth on these surfaces in different conditions also suggested that the two coatings possibly do not release toxic molecules that can compromise cell viability (Figure 5 and Table 1).

Spheroids have become advanced cell culture models that better mimic the in vivo microenvironment compared to 2D cultures. However, studying them presents several technical challenges and limitations. Studies carried out in our lab have demonstrated the applicability of 3D confocal profilometry as a simple and non-destructive method to assess surface features, including spheroids, with accuracy [25,33] (Figure 5). Compared to SHS1, spheroids in SHS2 are less uniform and compact, with greater variability in both size and shape. Despite these limitations, the trend of improved spheroid growth at higher fibroblast ratios remains consistent, reinforcing the role of fibroblasts in enhancing tumor spheroid architecture. However, the suboptimal performance of the 50:50 group and reduced overall compactness suggest that SHS2 conditions may impair fibroblast–carcinoma interactions or alter extracellular matrix dynamics critical for stable spheroid formation.

Given the similar values for the roughness provided by the same formulation nanoparticles, the different behavior can be explained by the chemistry of the SHS surfaces. In facts, the lower surface energy of the fluorinated sample (SHS1), allows a strong reduction of adhesion phenomena with respect to the silicone-based (SHS2) that show higher surface energy. In fluorinated systems, the higher hydrophobicity seems to allow for more spherical aggregates (ratio height/diameter) with respect to silicone-based systems where cells appear to be more adherent to the substrate, with lower growth in height (Figure 6). On SHS1, the spherical shape increases accordingly with cell ratio, showing a linear growth in height and slighter growth in diameter. On SHS2, height values are comparable in all the concentrations; the aggregates are likely a few layers of cells, showing the role of chemistry in letting the cells adhere to the surface. From height data, we can conclude that, in such cell ratios, aggregates do not have the shape and the size of a real spheroid, with these values being comparable to an order of magnitude of a cell bilayer [34,35].

Assuming that fibroblasts (3T3) actively secrete extracellular matrix proteins, and that carcinoma cells (A431) often respond to and remodel the ECM during tumor progression, variations in surface properties could indirectly or directly impact ECM dynamics and cell adhesion signaling. Works in the literature have demonstrated how for fibroblasts, surface cues may influence their ECM secretion patterns and organization, potentially creating microenvironments that vary in composition and mechanical properties. For carcinoma cells, which often exhibit enhanced integrin expression and altered adhesion signaling during transformation, these surface-induced ECM variations may further modulate their phenotypic behavior, including changes in motility and resistance to apoptosis, among others [36,37].

Generating well-defined, structurally stable, and viable spheroids is crucial for pharmaceutical applications, as variations in morphology, size, or cell viability can compromise the reproducibility and reliability of experimental outcomes. The physiological relevance of the proposed model is supported by its 3D architecture, as confirmed through 3D profilometry, and by the inclusion of fibroblasts, which significantly influence spheroid growth and viability. Accordingly, it is well established that larger or more compact spheroids often develop diffusion gradients, leading to hypoxic cores and limited nutrient and drug penetration [38]. Moreover, fibroblasts within tumor spheroids play a key role in tumor cell behavior by mimicking the tumor microenvironment. They promote tumor growth through paracrine signaling (e.g., TGF-β, FGF, EGF, HGF), activate proliferative pathways (MAPK, PI3K/AKT), and remodel the extracellular matrix to support expansion. Their spatial arrangement influences oxygen and nutrient distribution, further affecting cell proliferation and differentiation [39,40,41]. We believe that the generated structures could contribute to enhanced chemoresistance, thereby replicating critical aspects of the tumor microenvironment.

The stability of the SHS1 coating has been demonstrated in previous works and in different fields over a time window of few months even in day-night light cycles or in sea underwater conditions [42]. In biomedical applications it has been showed that they can reproduce the initial performance after a mild acid conditions treatment [43]. These features could significantly contribute to their spheroid-supportive properties and feasibility of scaling up for drug screening purposes. Collectively, these attributes underscore the broad utility of the spheroid model in advancing cancer research and expediting the development of more effective therapies.

## 4. Materials and Methods

### 4.1. Materials

To create the two types of superhydrophobic surfaces in both cases, fumed silica nanoparticles with primary particles size from 5 to 30 nm (EVONIK HDK H15) that were purchased from Degussa (Hannover, Germany), were used to obtain the desired roughness. One surface was produced by dispersing the nanoparticles in a commercial fluoropolymer blend containing a fluorosilane polymer (0.1 wt.%) in a hydrofluoroether solvent, for the second one silica nanoparticles were dispersed in a Ethyl Acetate (Sigma-Aldrich, St. Louis, MO, USA) solution containing commercial silicone (4 g/L) (Saratoga, Milan, Italy single component with acetic cross-linking use for assemblies and for general sealing).

High glucose DMEM medium (4.5 g/L glucose), L-glutamine solution (200 mM), fetal bovine serum (FBS), and penicillin–streptomycin solution (10,000 U/mL penicillin and 10 mg/mL streptomycin), were purchased from Lonza (Verviers, Belgium). Trypsin–EDTA solution (170,000 U/L trypsin and 0.2 g/L EDTA) and phosphate-buffered saline (PBS) were also purchased from Lonza. The 25 cm^2^ flasks and the culture dishes were obtained Corning (New York, NY, USA).

### 4.2. Methods

#### 4.2.1. Surface Preparation and Characterization

SH surfaces were both prepared by spray coting on glass substate. Fluorinated one was obtained using 2.0 g/L of the silica nanoparticles dispersion silicone one was obtaining spraying a silica/silicone dispersion where silica nanoparticles were 4 g/L. The resultant SHSs were examined to assess their high hydrophobicity and cleaned with deionized water to get rid of any physical adsorbed contaminants that might have an impact on cell viability. Surface wettability was studied through contact angle (CA) measurements at room temperature using an ASTRA view drop-shape tensiometer (developed at CNR-ICMATE [44] using MilliQ high purity grade water produced by an ion-exchange microfiltration system (Milli-Pore, Burlington, MA, USA). After gently placing 5 μL drops onto the surface, the CA was monitored until spreading equilibrium was reached. Additionally, the surface roughness (Sa) and structure of SH samples were assessed using 3D confocal and interferometric profilometry (Sensofar S-NEOX, Terrassa, Spain). 3D profilometry, because of its quick and non-destructive use and characterization in compliance with ISO 25178, it was selected to enable a large, scanned surface [45].

#### 4.2.2. Cell Cultures

Celltec UB provides the Murine Swiss albino fibroblasts (3T3) and the squamous cell carcinoma (A431) cell lines. Cells were grown in high-glucose DMEM medium supplemented with 10% (*v*/*v*) FBS, 1% (*v*/*v*) L-glutamine and 1% (*v*/*v*) antibiotic mixture at 37 °C and 5% CO_2_. Cells were cultured in 25 cm^2^ culture flasks and routinely split when cells were approximately 80% confluent.

#### 4.2.3. Cell Culture in Superhydrophobic Substrates

Cells were seeded by confining the required volume into delimited areas of the coated samples fixed by a Viton (fluorinated elastomers) O-ring, to avoid the sample floating in the Petri dish.

Cells were seeded at a final concentration of 2 × 10^6^ cells/mL. The non-tumoral/tumoral cell ratio was fixed to be 20:80, 50:50, and 80:20 (*v*/*v*). Cells were incubated for 24 and 48 h under 5% CO_2_ at 37 °C. The cell morphology and growth were monitored by optical microscopy through a Nikon inverted microscope equipped with a video camera (Moticam 1080 HDMI&USB, Moticam 1080 HDMI and USB, Motic Europe, Barcelona, Spain). The image processor (Motic Images 3.0 software, Moticam 1080 HDMI and USB (Motic Europe, Barcelona, Spain) was used to analyze the collected images.

#### 4.2.4. Fluorescence Microscopy Studies

To assist the formation of spheroids and the preserved viability of the involved cells, fluorescence microscopy using acridine orange/ethidium bromide (AO/EB) double staining was conducted [46]. Cells were seeded at the different cell ratios mentioned above into the coated glasses following the standard atmosphere, temperature and time conditions. After 48 h of incubation, the spent medium was eliminated, and the fluorescent dyes AO (0.5 μg/mL) and BE (10 μg/mL) were added. Fluorescence images were acquired with an Olympus BX41 microscope equipped with a UV-mercury lamp (100 W Ushio Olympus, Olympus Iberia, Barcelona, Spain) and a U-N51004v2-FlTC/TRITC-type filter set (FITC: BP480-495, DM500-545, BA515-535, and TRITC: BP550-570, DM575-, BA590-621). Images were digitized on a computer through a video camera (Olympus digital camera XC50, Olympus Iberia, Barcelona, Spain) and were analyzed with an image processor (Cell-B analysis).

#### 4.2.5. Profilometry Studies

After elimination of the spent medium, spheroids were fixed with 4% (*v*/*v*) paraformaldehyde for 15 min. Fixed cells in sterile PBS and low temperature (approximately 5 °C) were maintained until being scanned.

Confocal mode was used to analyze the morphology of the spheroids generated. Selected areas containing spheroids were scanned and the corresponding profiles analyzed with the SensoSCAN software 5.3 version (Sensofar, Terrassa, Spain). Morphological parameters (height and diameter) were analyzed as a function of the cell ratio and the SHS.

#### 4.2.6. Statistical Analyses

Prior to conducting statistical comparisons, all data were analyzed with the Levene test to assess homogeneity of variance and with the Shapiro–Wilk test to assess distribution. Homogeneous and normally distributed data were analyzed with parametric two-way analysis of variance (ANOVA) followed by Fisher’s least significant difference (LSD) post hoc test using GraphPad Prism^®^ software v.8 (GraphPad Software, Inc., La Jolla, CA, USA). Differences were considered significant at *p* < 0.05 (unless otherwise mentioned).

## 5. Conclusions

This study demonstrates that both surface chemistry and cell ratio composition critically influence spheroid formation in co-cultures of A431 carcinoma cells. The presence of fibroblasts (3T3 cells) plays a key role in regulating spheroid morphology, enhancing both size and structural uniformity. Higher fibroblast ratios promote more rapid and consistent spheroid formation, likely due to increased extracellular matrix deposition in a surface-dependent manner. Notably, the SHS1 surface significantly enhances 3D architecture and cell viability, enabling 3T3/A431 co-cultures to form well-defined spheroids. In contrast, SHS2 conditions are less conducive to forming tightly packed, uniform spheroids. These findings underscore the potential of the SHS1 substrate to tailor 3D cancer models and facilitate the study of tumor–stroma interactions in vitro, highlighting the critical roles of cellular composition and culture ratio in determining the quality of these models. Ongoing research is focused on endothelial-cancer cell interactions, as endothelial cells play a crucial role in tumor angiogenesis and invasiveness, making them a prime target for antitumor target.

## Figures and Tables

**Table 1:** Morphological parameters (circularity and size distribution) and estimated cell viability based on the green (AO) and red (EtBr) emission.

Surface	3T3/A431	Circularity (Mean ± SEM)		Size (Mean ± SEM)		Cell Viability
		24 h	48 h	24 h	48 h	48 h
SHS1	20:80	0.896 ± 0.018	0.900 ± 0.017	184 ± 22	153 ± 15	Green (viable) *●●○* Red (dead) *●●○*
	50:50	0.923 ± 0.018	0.945 ± 0.012	407 ± 39	354 ± 33	Green (viable) *●●●* Red (dead) *●○○*
	80:20	0.939 ± 0.011	0.931 ± 0.013	302 ± 21	422 ± 90	Green (viable) *●●●* Red (dead) *●○○*
SHS2	20:80	0.878 ± 0.019	0.899 ± 0.032	168 ± 40	230 ± 59	Green (viable) *●●○* Red (dead) *●●○*
	50:50	0.841 ± 0.064	0.887 ± 0.027	498 ± 75	500 ± 46	Green (viable) *●●●* Red (dead) *●○○*
	80:20	0.749 ± 0.068	0.953 ± 0.029	614 ± 63	520 ± 83	Green (viable) *●●○* Red (dead) *●●○*

●○○: low; ●●○: moderate; ●●●: high.

## Data Availability

Data are contained within the article.
